# Supplementary material for: A ferroelectric fin diode for robust non-volatile memory
Source: Nat Commun. 2024 Jan 13;15:513. doi: 10.1038/s41467-024-44759-5 (PMC10787831; doi:10.1038/s41467-024-44759-5)
Supplement: Supplementary file 1 — Supplementary Information [file 41467_2024_44759_MOESM1_ESM.pdf]

Supplementary Information for

A ferroelectric fin diode for robust non-volatile memory

Guangdi Feng[1,2,#], Qiuxiang Zhu[1,2,#], Xuefeng Liu[1], Luqiu Chen[1], Xiaoming Zhao[1], Jianquan Liu[1], Shaobing Xiong[1,3], Kexiang Shan[4], Zhenzhong Yang[1], Qinye Bao[1], Fangyu Yue[1], Hui Peng[1], Rong Huang[1], Xiaodong Tang[1], Jie Jiang[4], Wei Tang[5], Xiaojun Guo[5], Jianlu Wang[6], Anquan Jiang[7], Brahim Dkhil[8], Bobo Tian[1,2,*], Junhao Chu[1,3] & Chungang Duan[1,9]

[1]Key Laboratory of Polar Materials and Devices (MOE), Ministry of Education, Shanghai Center of Brain-inspired Intelligent Materials and Devices, Department of Electronics, East China Normal University, Shanghai 200241, China.

[2]Zhejiang Lab, Hangzhou 310000, China.

[3]Institute of Optoelectronics, Fudan University, Shanghai, 200433, China

[4]Hunan Key Laboratory of Super Microstructure and Ultrafast Process, School of Physics and Electronics, Central South University, Changsha 410083, China

[5]National Engineering Laboratory of TFT-LCD Materials and Technologies, Department of Electronic Engineering, Shanghai Jiao Tong University, Shanghai 200030, China

[6]Frontier Institute of Chip and System, Fudan University, Shanghai, 200433, China

[7]State Key Laboratory of ASIC & System, School of Microelectronics, Fudan University, Shanghai, 200433, China

[8]Université Paris-Saclay, CentraleSupélec, CNRS-UMR8580, Laboratoire SPMS, 91190 Gif-sur-Yvette, France.

[9]Collaborative Innovation Center of Extreme Optics, Shanxi University, Shanxi 030006, China.

[#]These authors contributed equally: Guangdi Feng, Qiuxiang Zhu.

[*]e-mail: bbtian@ee.ecnu.edu.cn

This PDF file includes:

Supplementary note 1

Figs. S1 to S23

Table 1

Supplementary Note 1: The programing of the FFD passive crossbar array.

A passive array architecture is appealing in for high packing density, but it suffers the sneak path current issue. The self-rectifying characteristic guarantees that only forward paths are allowed in the FFD passive array which will effectively inhibit the sneak paths*(1)*. It means that the target's conductance state can be "correctly" read out. A 2 × 2 FFD passive crossbar array is used to demonstrate the writing operations (**Fig. S17**). Under an external forward voltage, the FFD can be treated as a resistance. Under an external backward voltage, the FFD can be treated as a single ferroelectric capacitor because the backward conductance is extremely low. To illuminate the ferroelectric domain switching of a targeted FFD, it is treated as shunt-wound ferroelectric capacitor and resistance for a targeted unit and a single resistance for untargeted units under external forward voltages.

During a set operation (**Fig. S17a**), in the red sneak path, the device 2 (D2) and device 3 (D3) are forward biased while the device 4 (D4) is backward biased. D4 suffers most part of the external voltage since its huge resistance value under the backward voltage. The equivalent circuit can be simplified as **Fig. S17b** and **Fig. S17c** for the targeted D1 and the high voltage-biased D4 respectively. Note that two resistances (2R), D2 and D3 under a forward voltage bias, are connected to the ferroelectric capacitor (D4) in series (**Fig. S17c**). The series resistance plays a crucial role for the domain switching process since the domain-switching speed at V is limited by the maximum current flow through R_s in the circuit, where the V and R_s is the amplitude of the external voltage and series resistance respectively*(2)*. Considering that the $R_s = r$ in the target D1 circuit is more than 5 orders smaller than the $R_s = 2R+r$ in the D4 circuit, the quick programing process for the target D1 effect little on the domain configuration in D4.

During a reset operation (**Fig. S17d**), in the red sneak path, the D2 and D3 are backward biased while the D4 is forward biased. D2 and D3 together suffer most part of the external voltage since their huge resistance value under the backward voltage. If it satisfies that: $V_t/2 < V_c < V_t$, where the V_t and V_c are the external voltage and coercive voltage of the FFDs respectively, the V_t can only program the domain configuration in the targeted D1.

In summary, the diode characteristic and coercive voltage together enable the intended programing in the FFD passive crossbar array. The programmed ON (**Fig. S19a**) and OFF (**Fig. S19b**) conductance states, after triangular voltage wave of ±15 V, in 400 devices at cross points of alternate rows and alternate columns of the FFD passive crossbar array (**Fig. S18**) are carefully

checked one by one using a reading voltage of 3 V. These ON and OFF conductance states can be distinguished clearly with an ON/OFF ratio of ~ 10.

The detailed programing process in the 16×6 hardware ANN is presented in **Fig. S21a**. The conductance states are programed column by column. And to avoid the potential error operations, the set (odd steps) and reset (even steps) operations are performed successively. **Figure S21b** shows the final conductance distribution in the 16×6 hardware ANN.

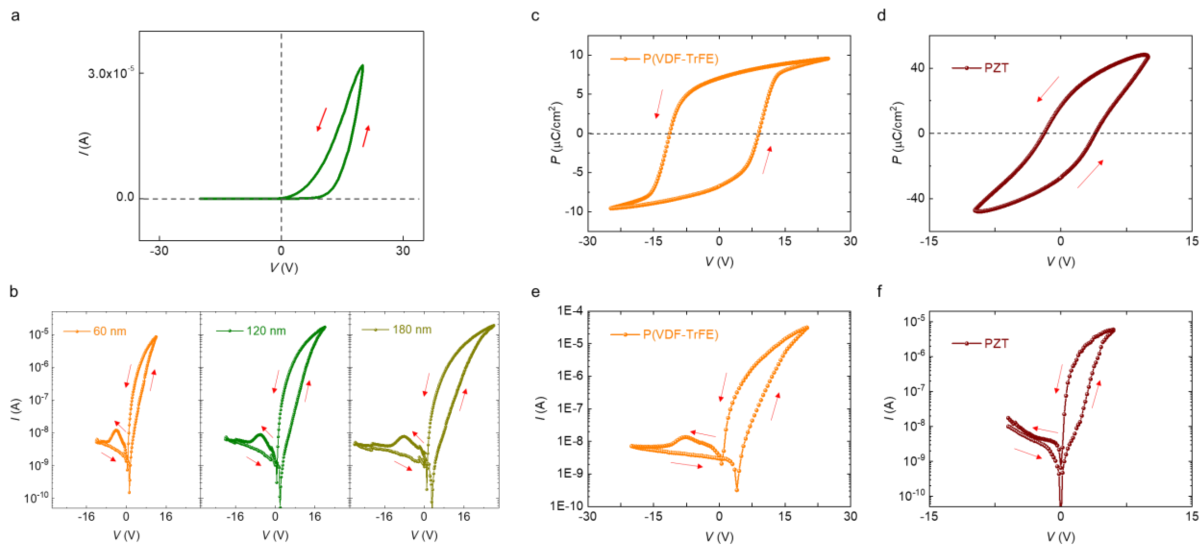

Fig. S1 (**a**) The quasi-static current versus voltage (*I*-*V*) curve in the linear coordinate for a FFD based on P(VDF-TrFE). **b.** The *I*-*V* curves of FFD device with a thickness of P(VDF-TrFE) layer being 60 nm, 120 nm, and 180 nm, respectively. (**c**-**d**) The *P*-*V* curves of FFD device based on 120 nm-thick P(VDF-TrFE) films (**c**) and 200 nm-thick PZT films (**d**), respectively. (**e-f)** The *I*-*V* curves of FFD device based on 120 nm-thick P(VDF-TrFE) films (**e**) and 200 nm-thick PZT films (**f**), respectively.

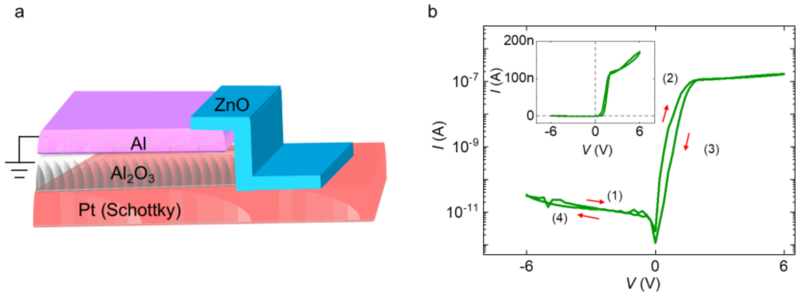

Fig. S2 (**a**) A dielectric fin diode based on Al_2O_3. (**b**) The quasi-static *I*-*V* curves of the dielectric fin diode.

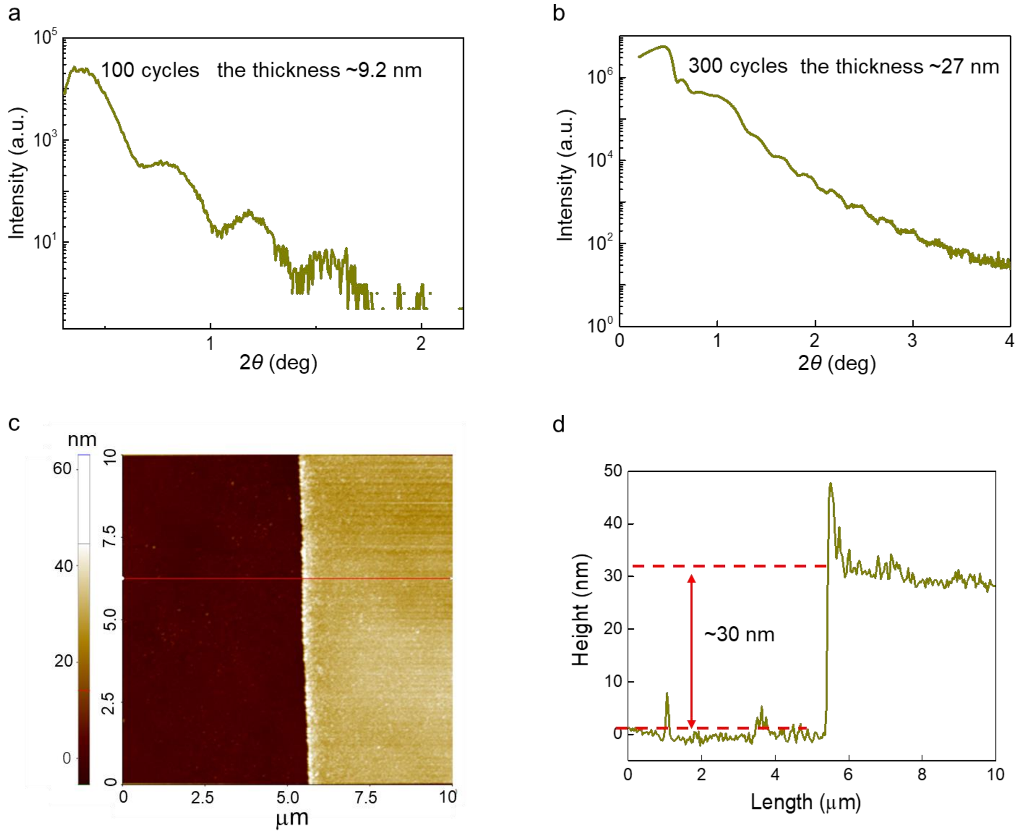

Fig. S3 (**a-b**) X-ray reflectivity (XRR) of as-deposited Al_2O_3 films with 100 cycles (**a**) and 300 cycles (**b**) using thermal atomic layer deposition (ALD). It gives a thickness of ~9.2 nm (27 nm) for Al_2O_3 films with 100 cycles (300 cycles) layer. (**c**) The height image of the ZnO step on a SiO_2/Si substrate using atomic force microscope (AFM). (**d**) The height data along the red line in (**c**). It gives a thickness of ~30 nm for ZnO layer. The ZnO layer was sputtered using same parameter as in main paper and the step is fabricated using standard photoresist lift off method.

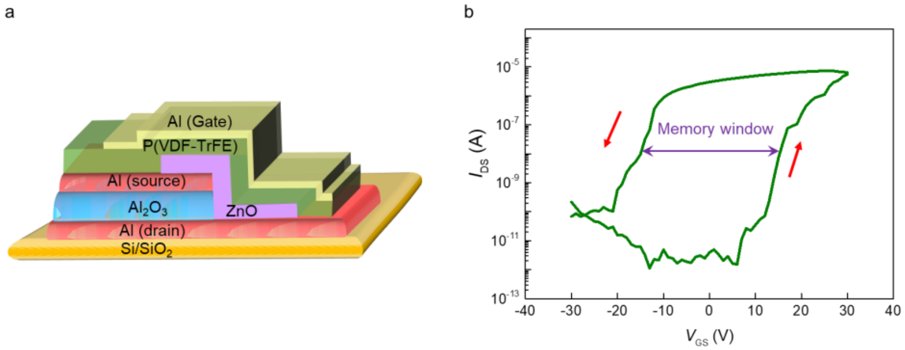

Fig. S4 (**a**) Schematic of standard vertical channel FeFET. (**b**) The quasi-static *I*-*V* curves of the vertical channel FeFET.

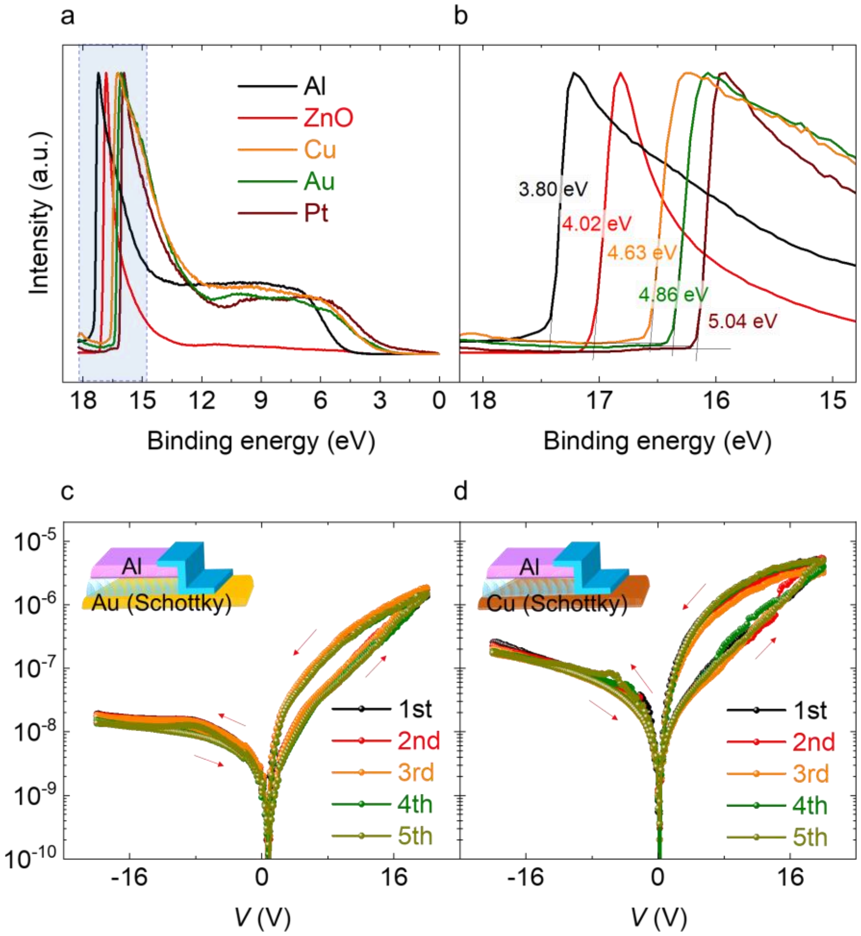

Fig. S5 (**a-b**) Ultraviolet photoelectron spectroscopy of Al, Cu, Au and Pt metals and ZnO semiconductor. (**b**) shows the detail in the region highlighted by blue dash frame in (**a**). The work function of Al, Cu, Au and Pt metals is obtained to be 3.80 eV, 4.63 eV, 4.86 eV and 5.04 eV respectively. An affinity of 4.02 eV is obtained in ZnO semiconductor. (**c-d**) The *I-V* curves of FFD device based on Au bottom electrode (**c**) and Cu bottom electrode (**d**) respectively.

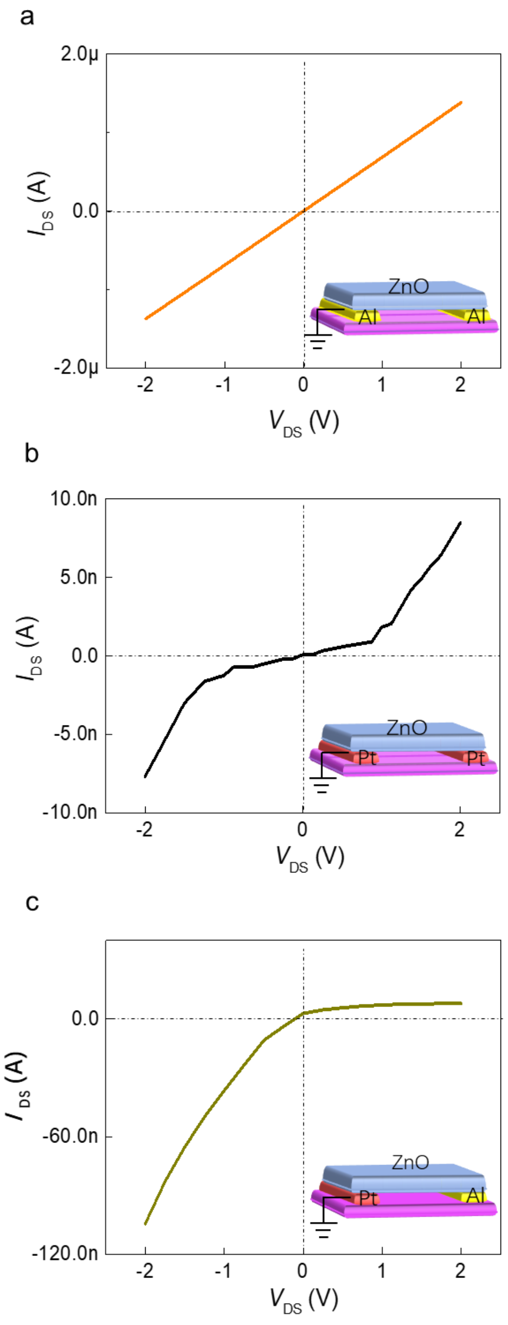

Fig. S6 (**a-c**) The output curve of ZnO transistor with different source/drain electrodes, Al/Al (**a**), Pt/Pt (**b**), and Al/Pt (**c**), respectively.

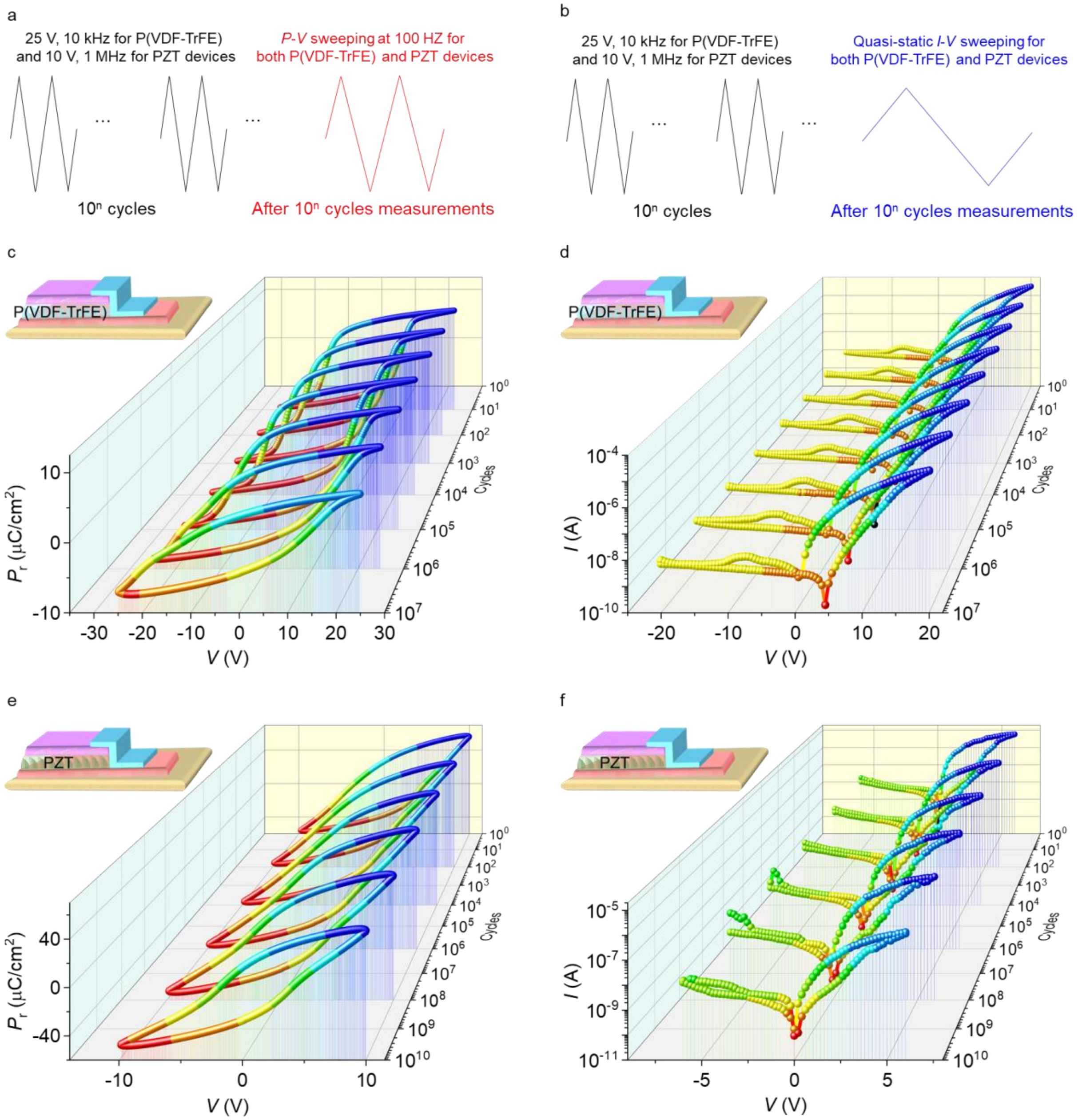

Fig. S7 (**a-b**) The schematic diagram of endurance measurements for remanent polarization (**a**) and resistive switching (**b**). (**c-d**) Evolution of *P-V* loops (**c**) and *I-V* curves (**d**) under endurance cycles in a typical FFD based on P(VDF-TrFE). (**e-f**) Evolution of *P-V* loops (**e**) and *I-V* curves (**f**) under endurance cycles in a typical FFD based on PZT.

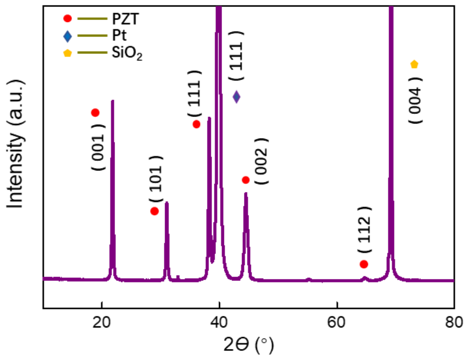

Fig. S8 The XRD pattern of the PZT films on Pt/SiO2/Si substrate.

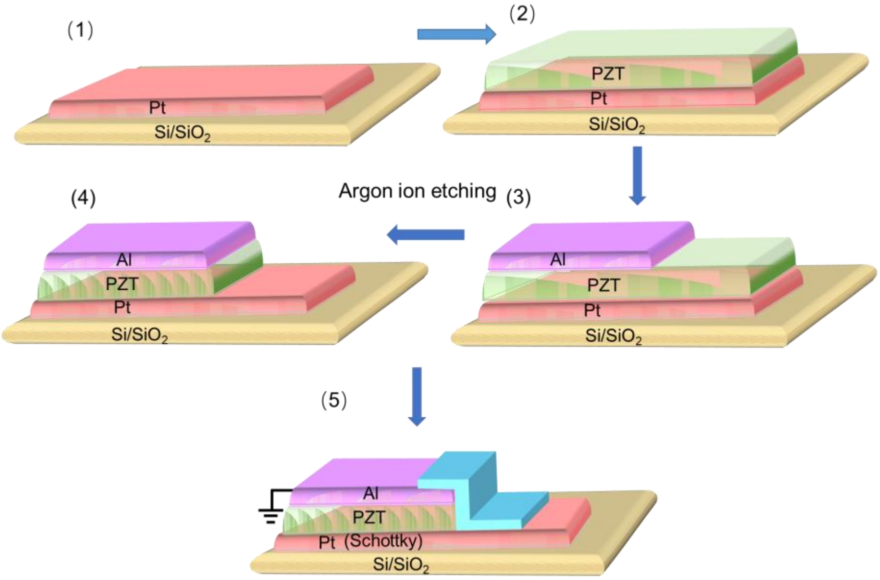

Fig. S9 **(1-5)** The fabrication process of the FFD based on PZT.

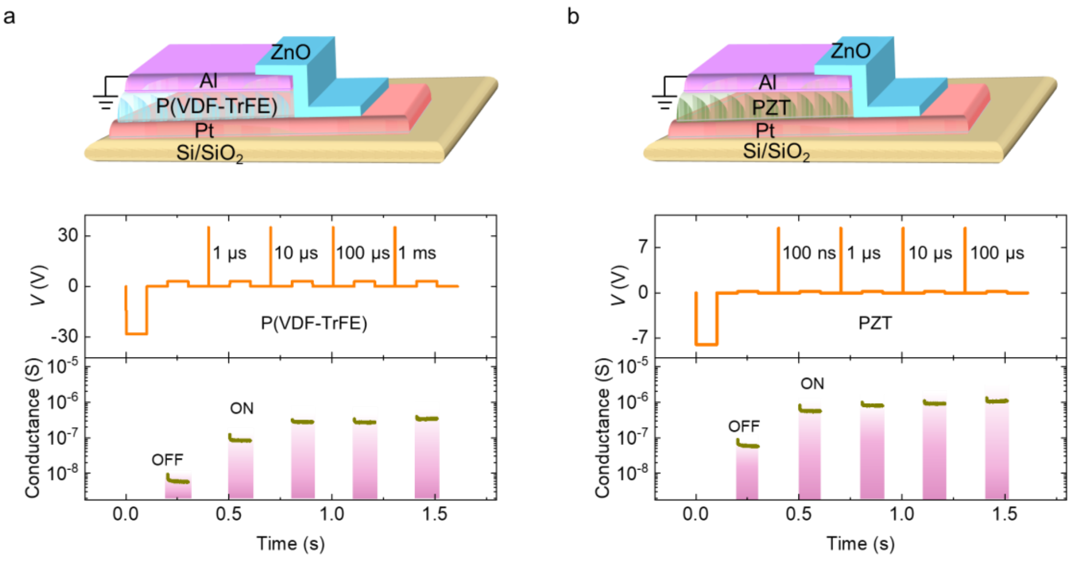

Fig. S10 (**a-b**) The programed voltage pulse sequence (top panel) and the evolution of conductance with time (bottom panel) for a typical FFD based on P(VDF-TrFE) (**a**) and PZT (**b**).

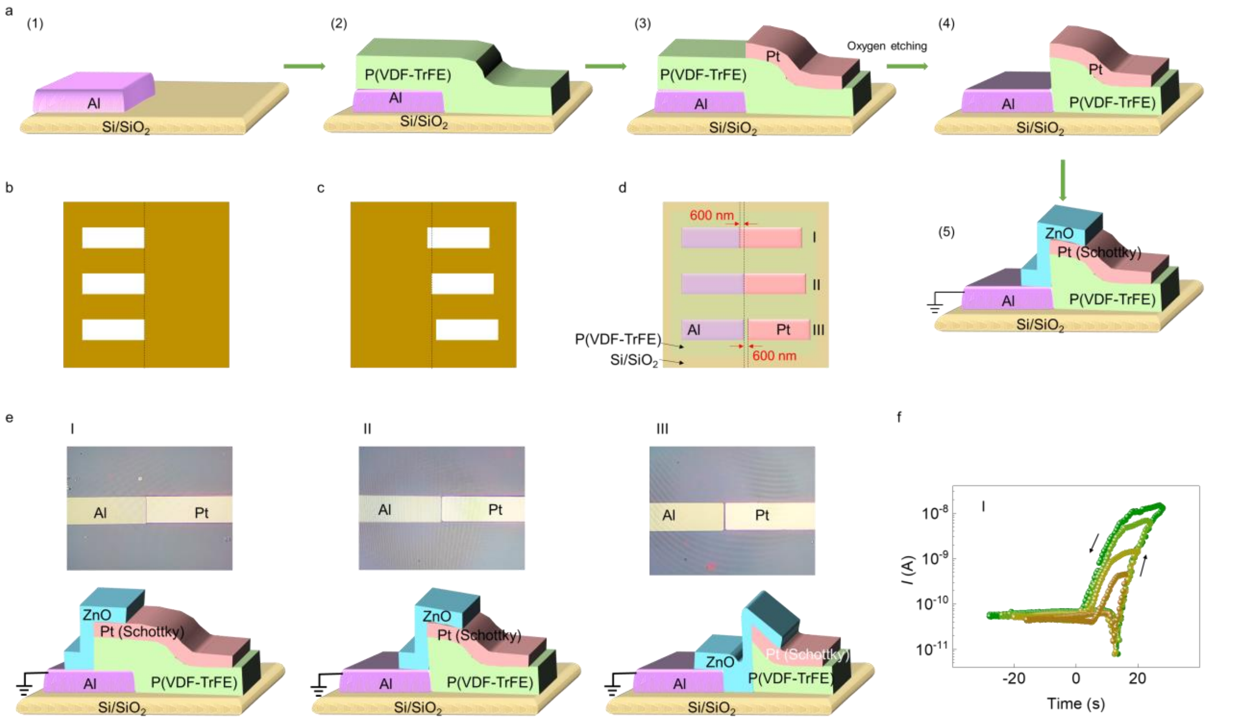

Fig. S11 (**a**) The fabrication process of a reversed FFD where the Pt electrode is on top of P(VDF-TrFE). (**b**) A mask with aligned patterns is used at stage (1) in (**a**) to photoetching Al bottom electrodes. (**c**) A mask with malposed patterns is used at stage (3) in (**a**) for photoetching Pt top electrodes. (**d**) The overlapped (I), “zero” overlapped (II) and separated (III) electrode

pairs of Al and Pt are formed simultaneously on a same sample substrate. (**e**) The optical images and device sketch of reversed devices with the overlapped (I), just “zero” overlapped (II) and separated (III) electrode pairs of Al and Pt. (**f**) The *I*-*V* curves of reversed FFD with overlapped (I) electrode pairs of Al and Pt. During the electrical measurements, the Al electrode is aways grounded. To avoid destroy of ferroelectricity during the spurring deposition of Pt electrode, thick P(VDF-TrFE) films with six spin-coating layers (~ 360 nm) are used in these reversed devices.

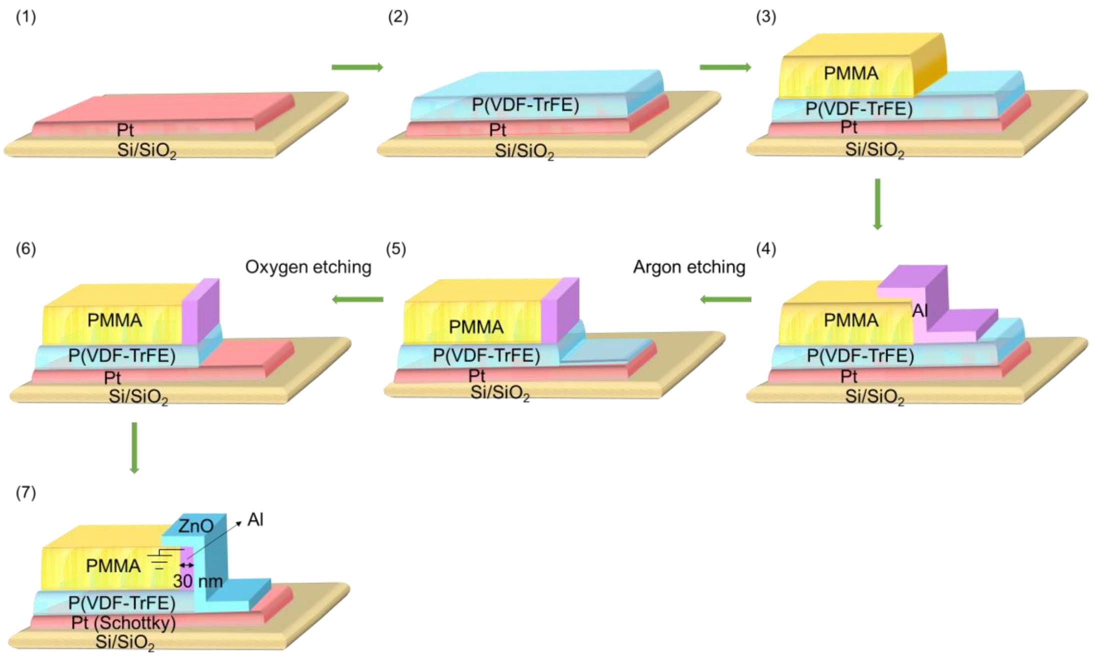

Fig. S12 **(1-5)** The fabrication process of FFD nano devices where the width of Al top electrode is only 30 nm. The thickness of P(VDF-TrFE) films in this FFD nano device is ~60 nm.

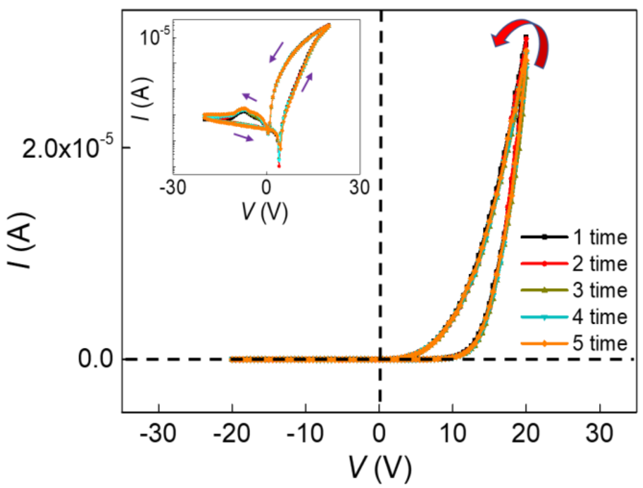

Fig. S13 The quasi-static *I*-*V* curves in a linear coordinate obtained during five continuous voltage sweepings on one unit device. Inset shows the *I*-*V* curves in a logarithmic coordinate.

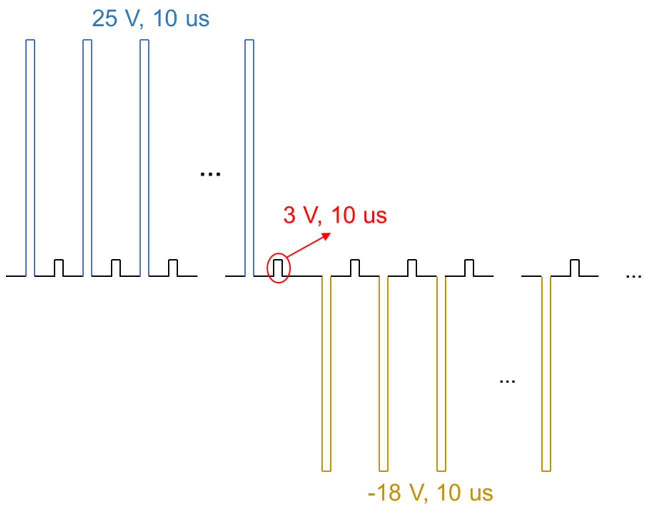

Fig. S14 The programed voltage pulse sequence of the quasi-linear conductance potentiation (strengthening) and depression (weakening) with 25 discrete states.

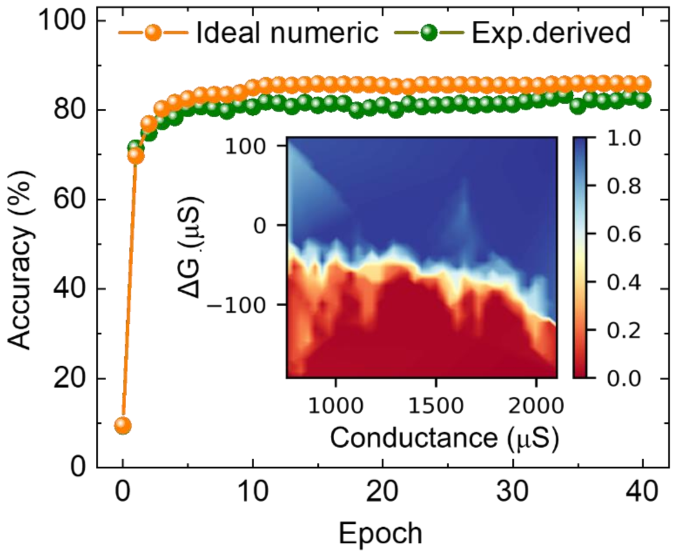

Fig. S15 Evolution of the accuracy with training epochs achieved by simulating the FFD-based artificial neural network for recognizing handwritten digits with 28×28 pixels. Insets show the probability distributions of the change in conductance (ΔG) induced by a write operation versus initial conductance at depression process.

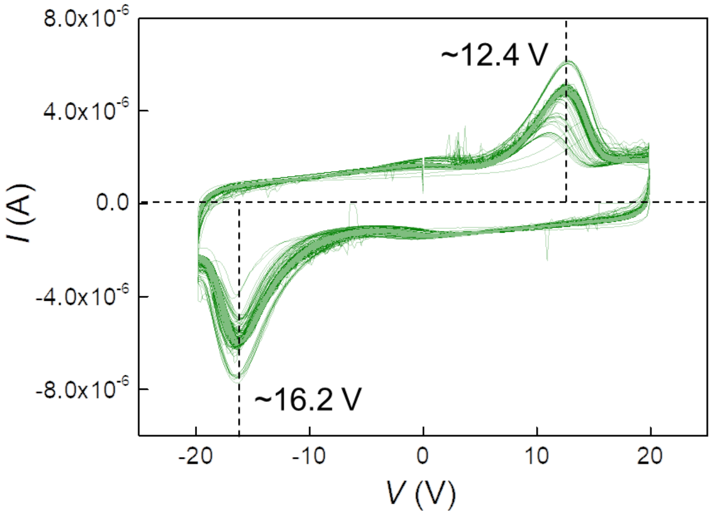

Fig. S16 The transient *I*-*V* curves at 200 Hz in 200 random-selected devices.

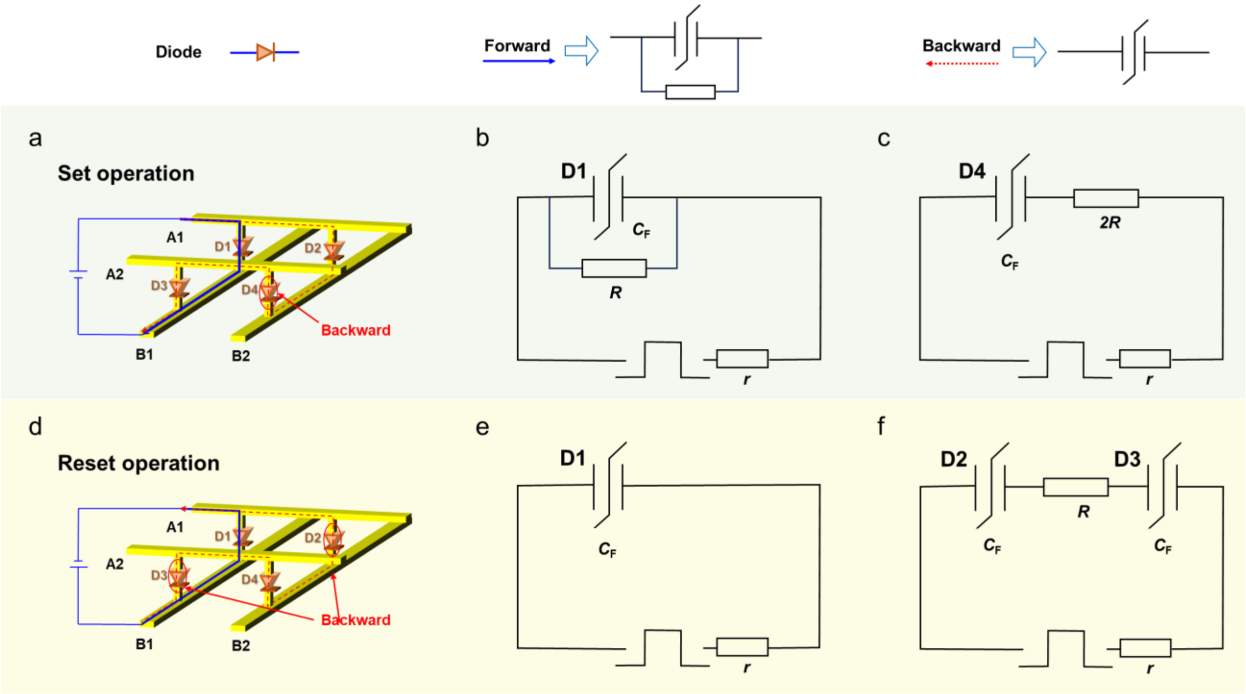

Fig. S17 The equivalent circuit for writing operations. (**a-c**), The equivalent circuit in a set operation for D1 (**b**) and D4 (**c**) from a 2 × 2 passive crossbar array (**a**). (**a-c**), The equivalent circuit in a reset operation for D1 (**e**) and D2 and D3 (**f**) from a 2 × 2 passive crossbar array (**d**).

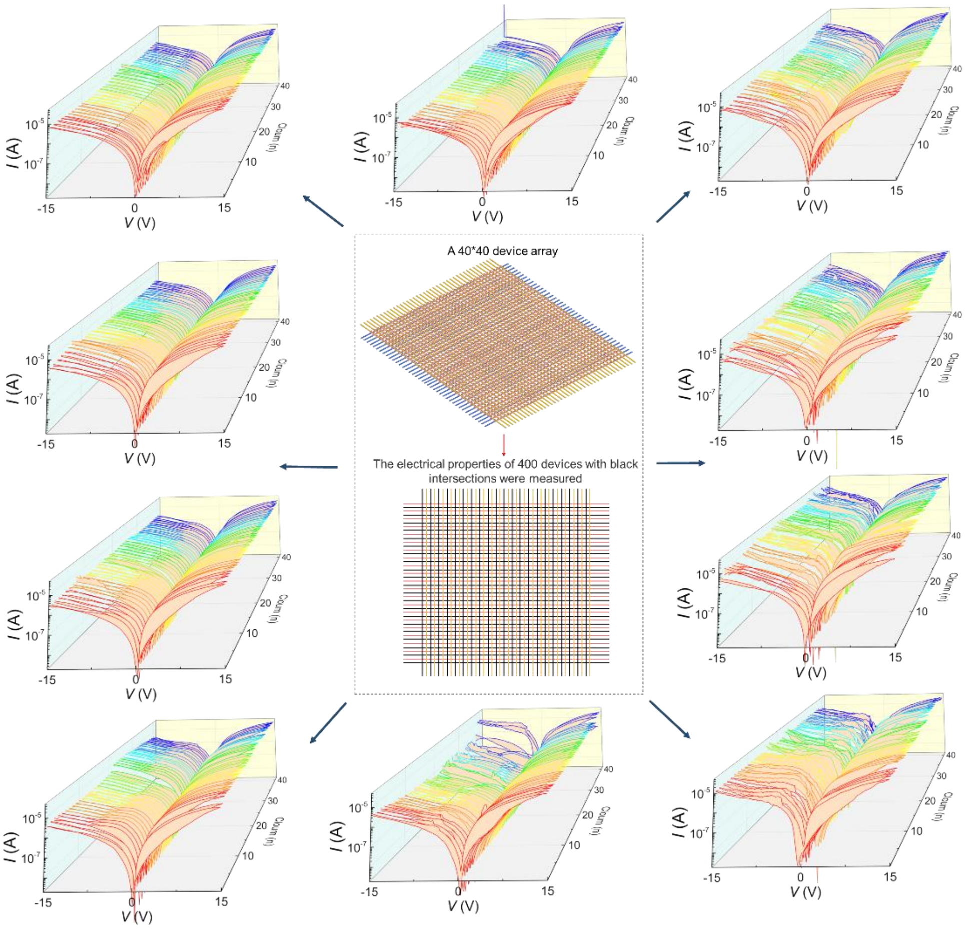

Fig. S18 *I*-*V* curves of FFD devices in a 40×40 passive crossbar array. To check the availability of this passive crossbar array, the resistive switching in 400 devices at cross points of alternate rows and alternate columns is carefully checked one by one.

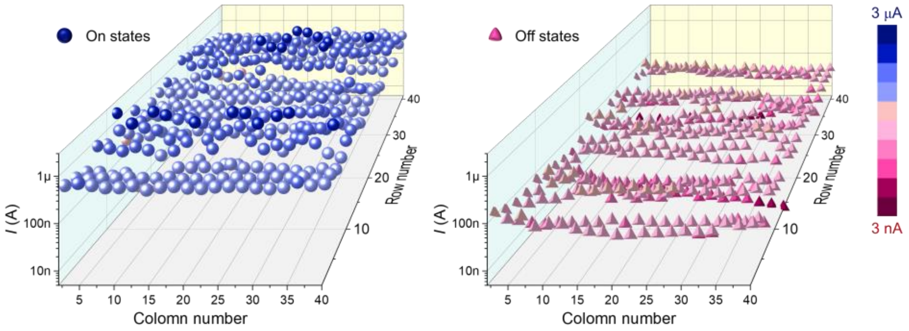

Fig. S19 (**a-b**), The programmed ON (**a**) and OFF (**b**) conductance states in 400 devices at cross points of alternate rows and alternate columns of the ferroelectric fin diode passive crossbar array.

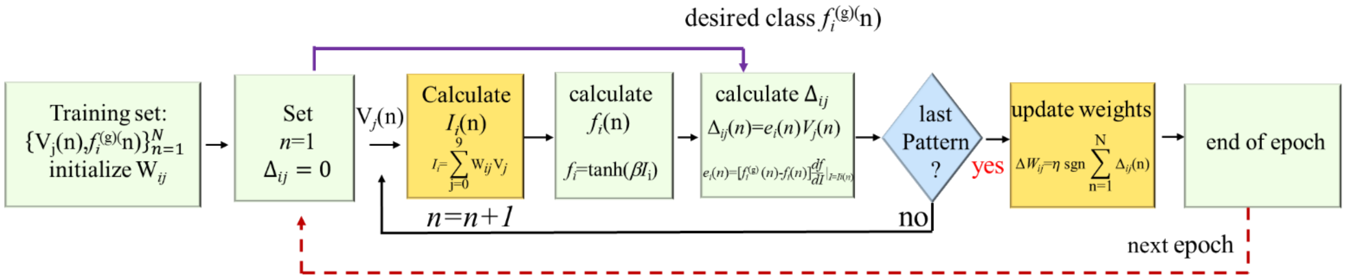

Fig. S20 The flow chart of training process basing on the Manhattan update rule.

.

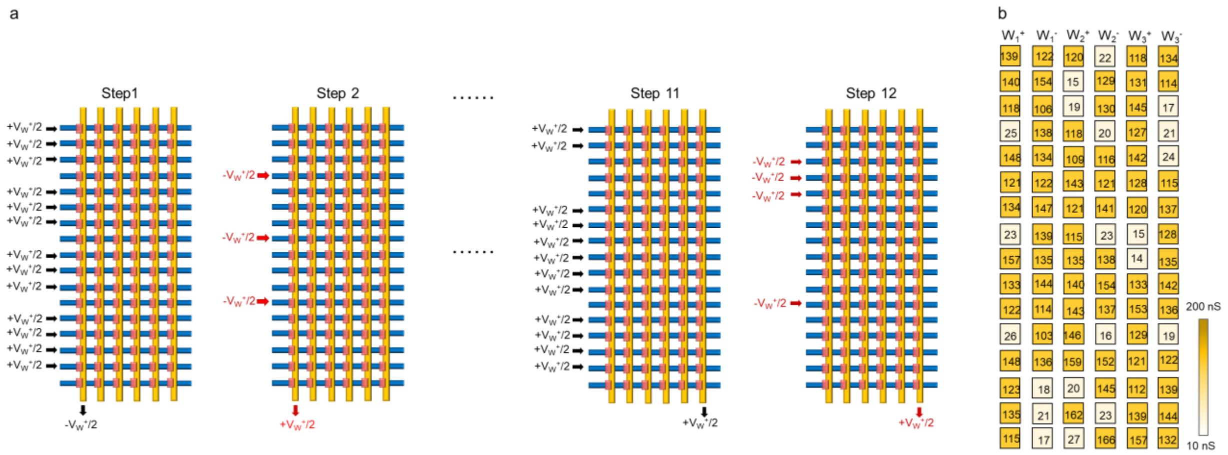

Fig. S21 (**a-b**) The programming process (**a**) and final conductance distribution (**b**) in the 16×6 hardware ANN. The half write voltage ($V_W/2$) is 6 V, the width of the write voltage pulse is 10 ms. The conductance distribution is checked using voltage pulses (1 V, 10 ms).

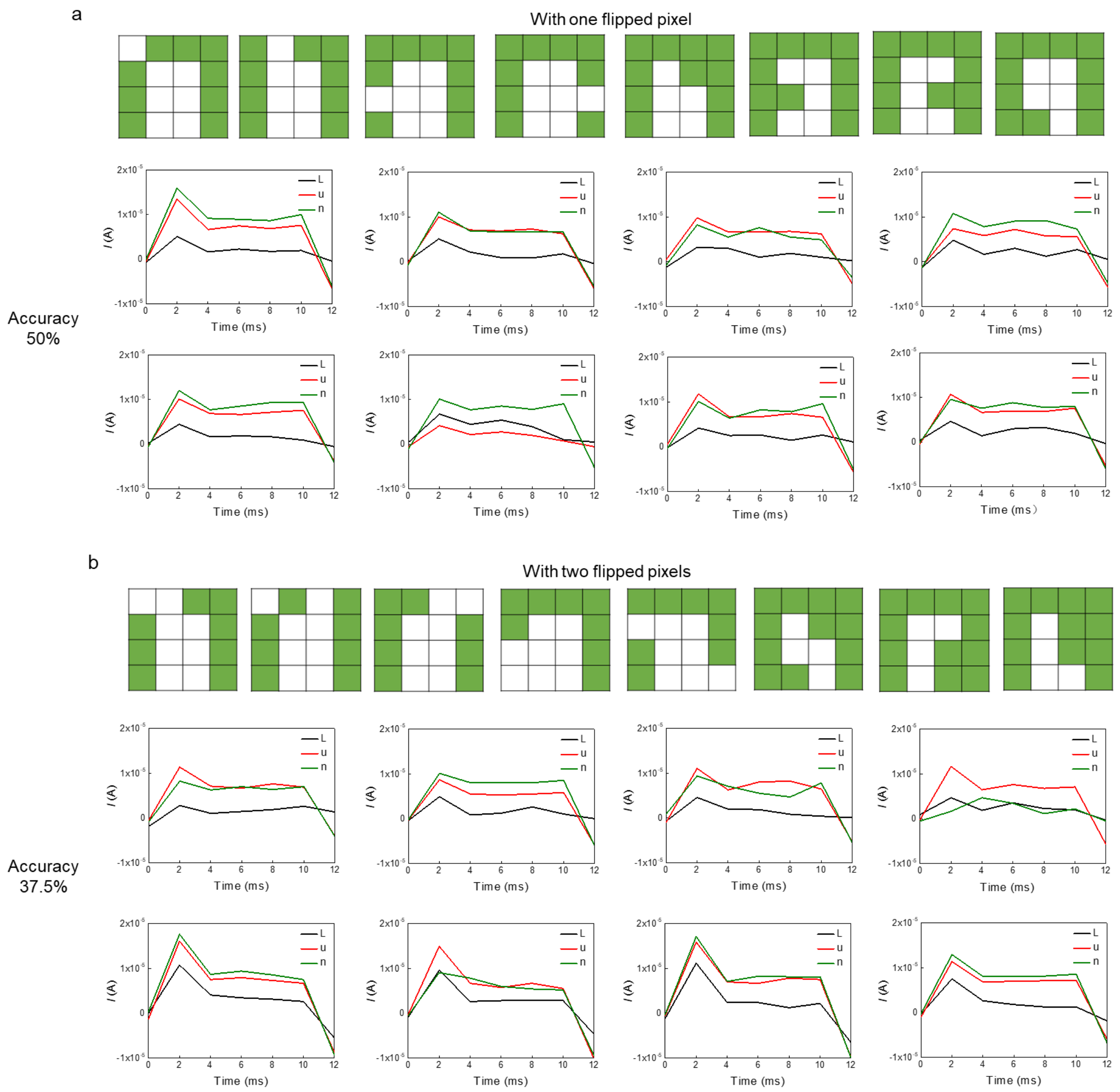

Fig. S22 (**a-b**) The current difference (I_n=I_n^+-I_n^-) collected from columns when “n” images with one flipped pixel (**a**) or two flipped pixels (**b**) are inputted into the 16×6 hardware ANN.

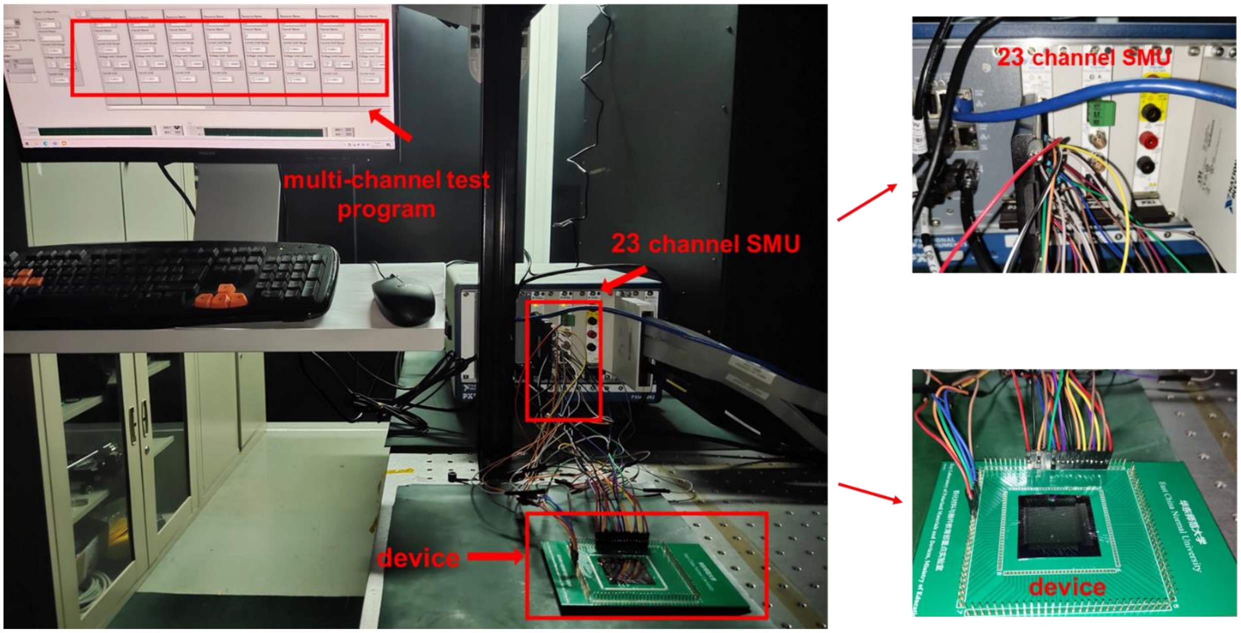

Fig. S23 Photograph of the measurement system for hardware ANN based on FFD devices.

Table S1. Performance comparison with state-of-the-art non-volatile memories*.

	NAND Flash	PCM	FeRAM	RRAM	MRAM	This work
Cell size	4/176L F^2	4/4L F^2	6 to 30 F^2	6 to 30 F^2	6 to 30 F^2	4 F^2
Energy	~ 0.01 pJ	~ 10 pJ	~ 0.1 pJ	~ 0.1 pJ	~ 0.1 pJ	~ 0.02 pJ
Speed	~ 10 µs	10 to 100 ns	10 to 100 ns	~ 100 ns	~ 10 ns	~ 100 ns
Endurance	~ 10^4	~ 10^7	~ 10^{15}	~ 10^6	~ 10^{15}	10^{10}
Rectification	1	1		1	1	10^4

Key parameters of state-of- the-art non-volatile memories including Not And logic gates (NAND Flash), phase change memory (PCM), FeRAM, resistive RAM (RRAM) and Magnetic RAM (MRAM) were obtained from recent review reports(3)*. Among the vast family of nonvolatile memories, this FFD cumulatively demonstrates very high performances with an endurance of over 10^{10} cycles, a self-rectification ratio of ~ 10^4, an operation speed of 100 ns, a feature size of 30 nm, and an ultralow energy consumption lower than 100 fJ.

References

1. L. Shi, G. Zheng, B. Tian, B. Dkhil, C. Duan, Research progress on solutions to the sneak path issue in memristor crossbar arrays. Nanoscale Adv. **2**, 1811–1827 (2020).

2. A. Jiang, H. J. Lee, C. S. Hwang, J. F. Scott, Sub-Picosecond Processes of Ferroelectric Domain Switching from Field and Temperature Experiments, Adv. Funct. Mater. **22**, 192–199 (2012).
3. M. Lanza, *et al.*, Memristive technologies for data storage, computation, encryption, and radio-frequency communication. *Science* **376**, eabj9979 (2022).
